# Accuracy of intravascular ultrasound-derived virtual fractional flow reserve (FFR) and FFR derived from computed tomography for functional assessment of coronary artery disease

**DOI:** 10.1186/s12938-023-01122-x

**Published:** 2023-06-27

**Authors:** Wenhao Huang, Jingyuan Zhang, Lin Yang, Yumeng Hu, Xiaochang Leng, Yajun Liu, Hongfeng Jin, Yiming Tang, Jiangting Wang, Xiaowei Liu, Yitao Guo, Chen Ye, Yue Feng, Jianping Xiang, Lijiang Tang, Changqing Du

**Affiliations:** 1 Department of Medicine, The Second College of Clinical Medicine, Zhejiang Chinese Medical University, Hangzhou, China; 2 Department of Geriatrics, The First Affiliated Hospital, Zhejiang University School of Medicine, Hangzhou, China; 3 ArteryFlow Technology Co., Ltd., Hangzhou, China; 4 Department of Cardiology, Zhejiang Hospital, Hangzhou, China; 5 Department of Radiology, Zhejiang Hospital, Hangzhou, China

**Keywords:** Coronary physiology, Fractional flow reserve, Intravascular ultrasound, Coronary computed tomography angiography, Diagnostic efficiency

## Abstract

**Background:**

Coronary computed tomography-derived fractional flow reserve (CT-FFR) and intravascular ultrasound-derived fractional flow reserve (IVUS-FFR) are two functional assessment methods for coronary stenoses. However, the calculation algorithms for these methods differ significantly. This study aimed to compare the diagnostic performance of CT-FFR and IVUS-FFR using invasive fractional flow reserve (FFR) as the reference standard.

**Methods:**

Six hundred and seventy patients (698 lesions) with known or suspected coronary artery disease were screened for this retrospective analysis between January 2020 and July 2021. A total of 40 patients (41 lesions) underwent intravascular ultrasound (IVUS) and FFR evaluations within six months after completing coronary CT angiography were included. Two novel CFD-based models (AccuFFRct and AccuFFRivus) were used to compute the CT-FFR and IVUS-FFR values, respectively. The invasive FFR ≤ 0.80 was used as the reference standard for evaluating the diagnostic performance of CT-FFR and IVUS-FFR.

**Results:**

Both AccuFFRivus and AccuFFRct demonstrated a strong correlation with invasive FFR (*R* = 0.7913, *P* < 0.0001; and *R* = 0.6296, *P* < 0.0001), and both methods showed good agreement with FFR. The area under the receiver operating characteristic curve was 0.960 (*P* < 0.001) for AccuFFRivus and 0.897 (*P* < 0.001) for AccuFFRct in predicting FFR ≤ 0.80. FFR ≤ 0.80 were predicted with high sensitivity (96.6%), specificity (85.7%), and the Youden index (0.823) using the same cutoff value of 0.80 for AccuFFRivus. A good diagnostic performance (sensitivity 89.7%, specificity 85.7%, and Youden index 0.754) was also demonstrated by AccuFFRct.

**Conclusions:**

AccuFFRivus, computed from IVUS images, exhibited a high diagnostic performance for detecting myocardial ischemia. It demonstrated better diagnostic power than AccuFFRct, and could serve as an accurate computational tool for ischemia diagnosis and assist in clinical decision-making.

## Introduction

The functional evaluation of coronary artery disease (CAD) plays a significant role in diagnosis and guiding treatment strategies in patients with known or suspected CAD. The invasive fractional flow reserve (FFR) has the highest recommendation (class IA) for the evaluation of CAD in societal guidelines [1]. However, the adoption of FFR in daily clinical practice is limited because of the invasive nature of the procedure, requirement of pressure wire, and the administration of hyperemic agents [2–4]. The computation of FFR from coronary artery imaging may increase the utility of FFR assessment in clinical practice. Recently, there has been increasing interest in these novel applications of FFR derived from coronary artery imaging without invasive pressure wire and administration of hyperemic agents [5].

Coronary artery imaging, such as coronary computed tomography angiography (CTA), intravascular ultrasound (IVUS), and optical coherence tomography (OCT), can provide anatomical information, including the arterial lumen structure and plaque characteristics. CTA, a non-invasive imaging method, has been recommended as a potential test before invasive coronary angiography (CAG) in the outpatient setting [6]. OCT and IVUS, which are both invasive imaging methods, offering higher resolution than CAG and CTA. Moreover, with anatomical information such as plaque characteristics, stent placement can be improved, and stent-related challenges can be minimized. However, anatomical information alone cannot reveal the functional significance for the target vessels. FFR derived from coronary artery imaging is a combination of anatimical and functional assessment.

In a previous study, a new method called AccuFFRivus was developed to calculate FFR using IVUS and CAG [7]. The method showed good diagnostic performance and strong correlation with FFR, indicating its potential for hybridizing coronary anatomical and physiological evaluation of CAD in the catheterization laboratory [7]. On the other hand, based on the computational fluid dynamics, CT-FFR can calculate the FFR from CTA [8]. It is a non-invasive technique for the functional assessment of coronary stenosis, allowing for a comprehensive coronary assessment outside the catheterization laboratory [9]. CT-FFR is recommended as the “gatekeeper” for coronary angiography and intervention, primarily in the outpatient setting [9–11]. Recently, a CT-FFR method called AccuFFRct was proposed, which can efficiently calculate non-invasive FFR based on anatomical and physiological information [12].

Both CT-FFR and IVUS-FFR can enable a one-stop assessment of the anatomical and functional aspects of CAD [13]. However, their computational algorithms and clinical utilization are quite different. Using FFR as the reference standard, this study aimed to compare the diagnostic performance between AccuFFRivus and AccuFFRct in real-world clinical practice.

## Results

### Baseline clinical and lesion characteristics

**Fig. 1:**
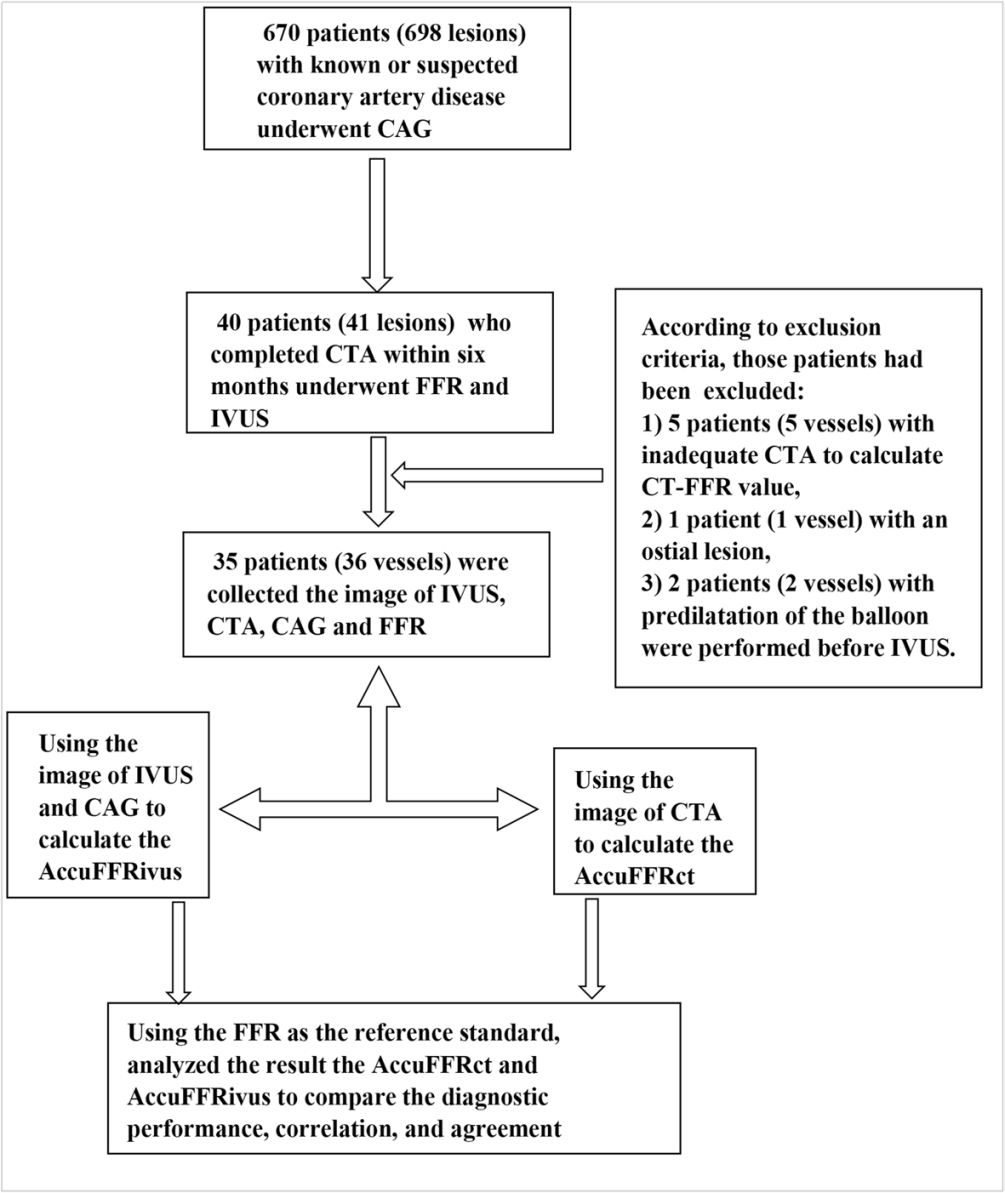
Participant flowchart of the study. *FFR* fractional flow reserve, *AccuFFRct* computed tomography-derived fractional flow reserve, *AccuFFRivus* intravascular ultrasound-derived fractional flow reserve, *CTA* computed tomography angiography, *CAG* coronary angiography, *IVUS* intravascular ultrasound

During the study period, 670 patients (698 vessels) with known or suspected CAD who had CAG were screened from January 2020 to July 2021. Among these patients, 59 patients (61 lesions) underwent both IVUS and FFR in our catheter lab. A total of 40 consecutive patients (41 lesions) who had the IVUS and FFR within six months after completing the CCTA were included. In the included population, five patients (five vessels) had inadequate CTA to calculate CT-FFR value, one patient (one vessel) had an ostial lesion, and two patients (two vessels) underwent predilatation of the balloon before IVUS. In the final analysis, 35 patients (36 vessels) were included in this study (Fig. 1). The mean patient age was 66.5 ± 8.3 years, and 29 (82.8%) were men. Approximately 65.7% of patients had a history of smoking. The mean left ventricular ejection fraction was 64.2 ± 8.67%. The baseline clinical patient characteristics are listed in Table 1. The target vessels included 24 (66.7%) left anterior descending arteries, five (13.9%) left circumflex arteries, and seven (19.4%) right coronary arteries. CTA-derived DS% ≥ 50% was observed in 28 (77.8%) vessels. The mean values of AccuFFRivus, AccuFFRct, and FFR were 0.72 ± 0.10, 0.74 ± 0.09, and 0.73 ± 0.09, respectively. The mean of the intravascular ultrasound-derived minimum lumen area (IVUS-MLA) was 2.83 ± 0.53 mm^2^. The baseline lesion characteristics is listed in Table 2.

**Table 1:** Baseline clinical patient characteristic

Patient characteristics	Number of patients (35)
Age, years	66.5 ± 8.33
Male	29 (82.8%)
Hypertension	33 (94.3%)
Diabetes mellitus	17 (48.6%)
Smoking history	23 (65.7%)
Drinking history	24.12 ± 2.34
BMI, kg/m2	17 (48.6%)
Family history of CAD	0 (0%)
Previous CABG	6 (17.1%)
Previous PCI	3 (8.6%)
Clinical presentation	
Stable angina	16(44.4%)
Unstable angina	6(16.6%)
Echocardiographic data	
Left ventricle ejection fraction (%)	64.2 ± 8.67
Left ventricle internal dimension (mm)	3.12 ± 0.39
Left ventricle diastolic diameter (mm)	4.83 ± 0.48

Values are mean ± SD or *n* (%)

*BMI* body mass index, *CAD* coronary artery disease, *CABG* coronary artery bypass grafting, *PCI* percutaneous coronary intervention, *MI* myocardial infarction

**Table 2:** Vessel and lesion characteristic

Vessel and lesion characteristics	Number of vessels (36)
2-D QCA feature	
Diameter stenosis, %	68.8 ± 10.6
Vessel	
LAD	24 (66.7%)
LCX	5 (13.8%)
RCA	7 (19.4%)
CTA feature	
Diameter stenosis ≥ 50%	28 (77.8%)
Cardiac calcium scoring scan	
< 100	22 (61.1%)
100–400	10 (27.8%)
> 400	4 (11.1%)
IVUS features	
IVUS-derived RVD (mm)	3.00 ± 0.36
IVUS-derived MLD (mm)	1.75 ± 0.27
IVUS-derived MLA (mm^2^)	2.83 ± 0.53
Area stenosis (%)	56.0 ± 9.0
Plaque burden (%)	73.6 ± 10.1
Functional indexes	
AccuFFRivus	0.72 ± 0.10
AccuFFRct	0.74 ± 0.09
FFR	0.73 ± 0.09
FFR ≤ 0.80	29 (80.6%)

*LAD* left anterior descending coronary artery, *LCX* left circumflex artery, *RCA* right coronary artery, *MLD* minimum lumen diameter, *MLA *minimum lumen area, *RVD* reference vessel diameter, *RVA* reference vessel area, *FFR *fractional flow reserve, *AccuFFRct* computed tomography-derived fractional flow reserve, *AccuFFRivus* intravascular ultrasound-derived fractional flow reserve

### Comparison of the correlations and agreements among AccuFFRivus, AccuFFRct, and invasive-FFR

**Fig. 2:**
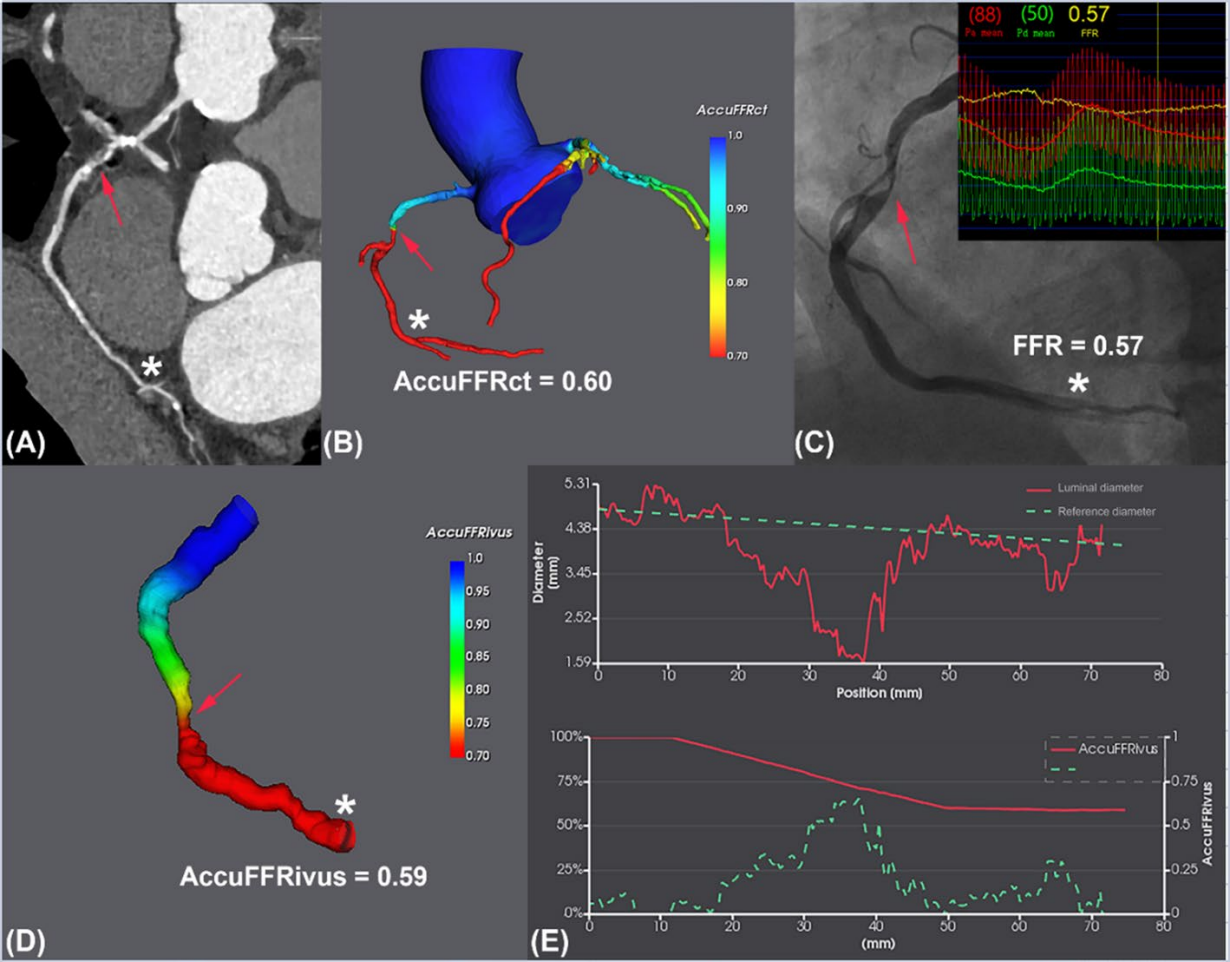
A representative example of AccuFFRct, AccuFFRivus, and FFR measurements. **A** Computed tomography angiography (CTA)-derived percentage diameter stenosis (%DS) = 60%. **B** Fractional flow reserve derived from computed tomography (AccuFFRct) = 0.60. **C** Wire-based fractional flow reserve (FFR) = 0.57. **D** Fractional flow reserve derived from intravascular ultrasound (AccuFFRivus) = 0.59. **E** Lumina diameter and AccuFFRivus pull back. Red arrows indicate the stenosis lesion. Asterisks indicate the location of measurement

**Fig. 3:**
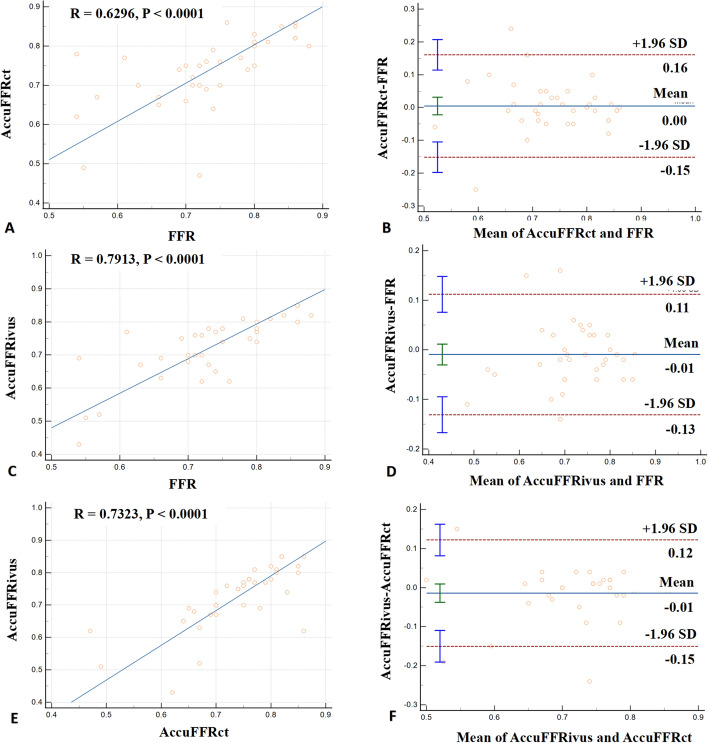
Correlations and agreements among AccuFFRivus, AccuFFRct, and FFR. **A** Correlation between AccuFFRct and FFR. **B** Agreement between AccuFFRct and FFR. The mean value of AccuFFRct-FFR = 0.00, the upper limit of agreement = 0.16, and the lower limit of agreement = − 0.15. **C** Correlation between AccuFFRivus and FFR. **D** Agreement between AccuFFRivus and FFR. Mean value of AccuFFRivus-FFR = − 0.10, the upper limit of agreement = 0.11, and the lower limit of agreement = − 0.13. **E** Correlation between AccuFFRivus and AccuFFRct. **F** Agreement between AccuFFRivus and AccuFFRct. The mean value of AccuFFRivus-AccuFFRct = − 0.01, the upper limit of agreement = 0.12, and the lower limit of agreement = − 0.15

Figure 2 illustrates a visual representation of AccuFFRivus, AccuFFRct, and invasive-FFR measurements. Figure 3 presents the correlation and agreement among these measurements. The results demonstrate that both AccuFFRivus and AccuFFRct are strongly correlated with invasive FFR, with R of 0.7913 (*P* < 0.0001) and 0.6296 (*P* < 0.0001), respectively. A good agreement is demonstrated by both AccuFFRivus and AccuFFRct with invasive-FFR, with similar mean differences of − 0.0094 ± 0.061 and 0.0050 ± 0.080, respectively. Additionally, a high correlation was observed between AccuFFRivus and AccuFFRct (*R* = 0.7323, *P* < 0.0001), and moderate agreement between the two measurements, with a mean difference of − 0.0144 ± 0.069 (Fig. 3).

### Diagnostic performance of AccuFFRivus and AccuFFRct for predicting FFR ≤ 0.80

**Fig. 4:**
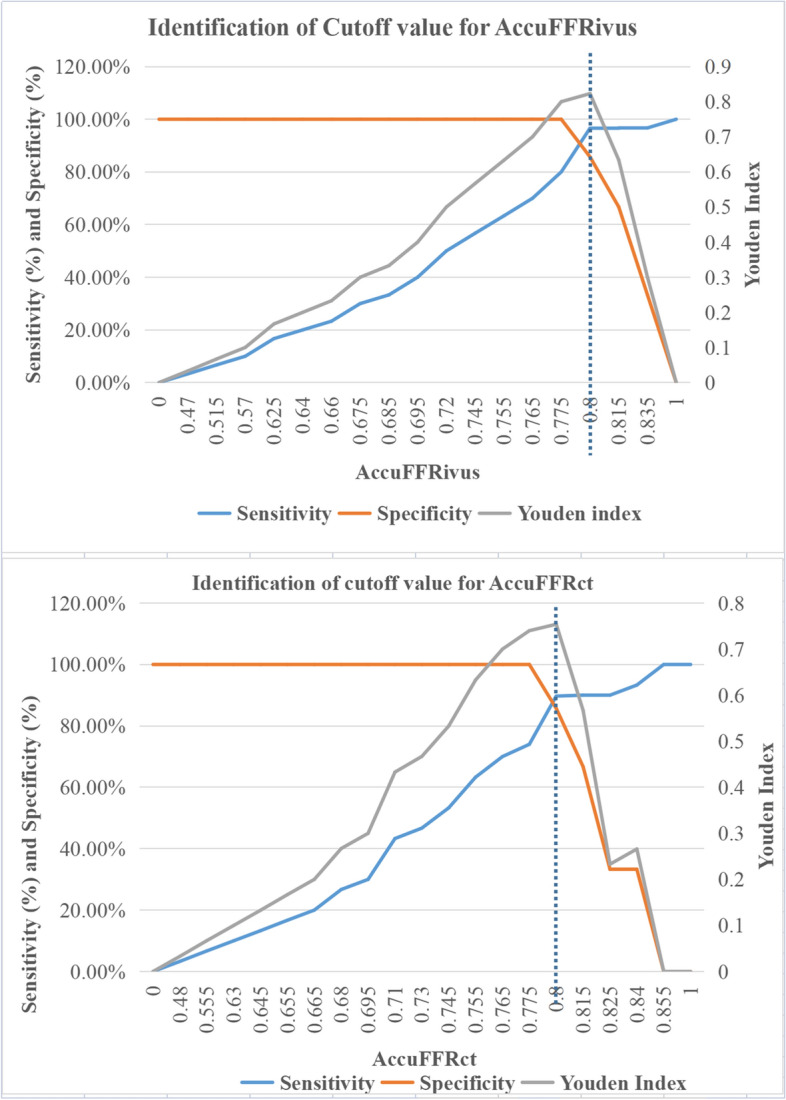
Sensitivity specificity and Youden index for different cutoff values of AccuFFRivus and AccuFFRct to predict FFR ≤  0.80. **A** The optimal AccuFFRivus cutoff value for predicting FFR ≤ 0.80 was 0.80 (sensitivity 96.6%, specificity 85.7%, Youden index 0.823). **B** The optimal fractional AccuFFRct cutoff value for predicting FFR ≤ 0.80 was 0.80 (sensitivity 89.7%, specificity 85.7%, Youden index 0.754)

**Fig. 5:**
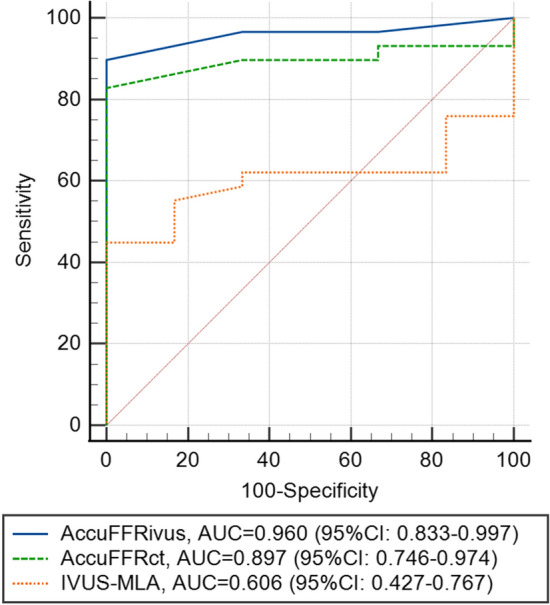
The receiver operating characteristic curves of AccuFFRivus, AccuFFRct, and IVUS-MLA in detecting FFR ≤ 0.80. IVUS-MLA: the minimum lumen area derived from intravascular ultrasound. *AccuFFRct* computed tomography-derived fractional flow reserve, *AccuFFRivus* intravascular ultrasound-derived fractional flow reserve

Figure 4 illustrates the sensitivity, specificity, and Youden index for different cutoff values of AccuFFRct and AccuFFRivus in predicting FFR ≤ 0.80. The optimal cutoff value for AccuFFRivus and AccuFFRct to predict FFR ≤ 0.80 was 0.80 and 0.80, with a sensitivity of 96.6% and 89.7%, specificity of 85.7% and 85.7%, and a Youden index of 0.823 and 0.754, respectively. Notably, AccuFFRivus demonstrated a much better diagnostic performance in detecting ischemia-causing stenoses than that of AccuFFRct. Figure 5 presents the receiver operating characteristic (ROC) curves for AccuFFRivus, AccuFFRct, and IVUS-derived MLA for FFR ≤ 0.80 predictions. The AUC for AccuFFRivus was significantly higher than that for IVUS-derived MLA (0.960 vs. 0.606) and AccuFFRct (0.960 vs. 0.897). This also demonstrated the superior diagnostic ability of AccuFFRivus in identifying whether stenoses can lead to ischemia.

### Diagnostic performance of AccuFFRivus ≤ 0.80 and AccuFFRct ≤ 0.80 for predicting FFR ≤ 0.80

The diagnostic performance of AccuFFRivus ≤ 0.80 and AccuFFRct ≤ 0.80 for predicting FFR ≤ 0.80 is presented in Table 3. Among all 36 vessels, using FFR as the reference standard, AccuFFRivus had 28 true positives (TP), six true negatives (TN), one false positive (FP), and one false negative (FN), while AccuFFRct had 26 TP, 6 TN, 1 FP, and 3 FN. AccuFFRivus had a false discovery rate of 3.4% (positive predict value 96.6%) and a false omission rate of 14.3% (negative predict value 85.7%) compared with FFR. This indicates that 5.6% of stenoses were misclassified using AccuFFRivus compared to FFR. On the other hand, the AccuFFRct also showed a relatively good diagnostic performance, 11.1% were misclassified using AccuFFRct compared to FFR. The diagnostic accuracy of AccuFFRivus ≤ 0.80 for predicting FFR ≤ 0.80 was 94.4% (95% confidence interval [CI]: 95.5–99.9%), while that of AccuFFRct was 88.9% (95% CI: 67.2–93.6%). A good diagnostic performance indicates that an accurate assessment of coronary stenosis is feasible.

**Table 3:** Diagnostic performance of AccuFFRivus and AccuFFRct  for predicting FFR ≤ 0.80

	AccuFFRivus ≤ 0.80	AccuFFRct ≤ 0.80
Accuracy, %	94.4 (81.3–99.3)	88.9 (73.9–96.9)
Sensitivity, %	96.6 (82.2–99.9)	89.7 (72.7–97.8)
Specificity, %	85.7 (42.1–99.6)	85.7 (42.1–99.6)
Positive predictive value, %	96.6 (82.0–99.4)	96.3 (80.8–99.4)
Negative predictive value, %	85.7 (46.1–97.7)	66.7 (39.7–85.9)
Positive likelihood ratio	6.76 (1.10–41.54)	6.28 (1.02–38.69)
Negative likelihood ratio	0.04 (0.01–0.28)	0.12 (0.04–0.37)

Values are n (95% confidence interval). Abbreviations as in Table 2

## Discussion

FFR was used as the reference standard in the present study to assess the diagnostic performance of AccuFFRivus and AccuFFRct. The primary study findings are summarized as follows: (a) both AccuFFRct and AccuFFRivus demonstrated strong correlations and good agreements with FFR. (b) The AUC of AccuFFRivus demonstrated better discrimination ability than AccuFFRct in defining hemodynamic significance of coronary stenosis. (c) The diagnostic performance of AccuFFRivus is better than that of AccuFFRct. (d) Compared to AccuFFRct, AccuFFRivus allows for simultaneous intracoronary imaging and functional assessment of coronary artery lesions in the cardiac catheterization laboratory, showcasing its potential in the coronary anatomical and physiological evaluation of CAD. This unique capability highlights that by integrating anatomical and functional information, AccuFFRivus provides a more comprehensive assessment of CAD.

Although CTA has become an essential tool for assessing the morphological features of coronary arteries in patients with CAD, it has limitations in determining whether coronary artery stenosis is the underlying cause of myocardial ischemia [14]. To address this limitation, CT-FFR has emerged as a non-invasive method to evaluate the functional significance of coronary arteries. Several early prospective, large-scale, multicenter studies comparing CT-FFR with invasive FFR have demonstrated the diagnostic efficacy of CT-FFR [15–18]. In our study, AccuFFRct incorporates three-dimensional (3D) reconstruction of coronary artery geometry and patient-specific physiological parameters including blood pressure, heart rate [12]. When comparing the diagnostic performance of AccuFFRct to invasive-FFR, AccuFFRct demonstrated good diagnostic accuracy in assessing the functional relevance of target vessels (AUC = 0.897, accuracy 88.9%). Thses results  were comparable to previous studies such as DISCOVER-FLOW (per-vessel AUC = 0.90, accuracy 84.3%), NXT (per-vessel AUC = 0.93, accuracy 86%) and MACHINE (AUC = 0.84, accuracy 73%). Additionally, the correlation (*R* = 0.6296) and diagnostic performance of AccuFFRct in the present study were similar to the previous AccuFFRct study (per-patient AUC = 0.945, per-vessel AUC = 0.925, per-patient *R* = 0.76, per-vessel *R = *0.65, accuracy = 90.7%). Notably, the computational time for AccuFFRct (35 min) was significantly shorter than previous studies, such as DISCOVER-FLOW (5 h) and NXT (1–4 h). A critical factor for patients with CAD during diagnosis is time efficiency; therefore, it is vital to shorten the diagnostic time to assist patients in making timely clinical decisions and therapeutic regimens. AccuFFRct offers  a time-efficient and accurate tool for FFR computation, which can be used for the early screening of CAD. CT-FFR has been recommended as the “gatekeeper” to the pathway of coronary angiography in the outpatient setting by improving detection accuracy, shortening time, and reducing the cost [9–12, 15–18]. The adoption of AccuFFRct can further enhance the role of CT-FFR in streamlining the diagnostic process for CAD, providing a valuable and efficient tool for clinicians and patients alike.

For the functional assessment of coronary artery lesions, IVUS-FFR is being developed as an alternative approach to CT-FFR. Previous studies have explored the feasibility of using computational fluid dynamics (CFD) to simulate FFR and improve diagnostic accuracy. Carrizo et al. reconstructed a coronary LAD from IVUS images and used CFD to calculate the fractional flow reserve [19]. In a subsequent study, including 24 patients (34 vessels), IVUS-FFR demonstrated better accuracy, sensitivity, and specificity in detecting ischemia compared to MLA or angiography. However, CFD simulations required the target vessel reconstruction for each arterial branch and took an average of 9.1 h per study vessel [20]. Seike et al. used FFR ≤ 0.80 as the diagnostic gold standard, and a strong correlation was found between IVUS-FFR and FFR (*R* = 0.78), higher than that between IVUS-MLA and FFR (*R* = 0.43) [21]. Similarly, Wei et al. reported a strong correlation between IVUS-FFR and FFR (*R* = 0.87) using invasive FFR ≤ 0.80 as the gold standard, with an AUC of 0.97, which was higher than that of IVUS-MLA (0.89) [22]. No significant differences were found between IVUS-FFR and FFR , regardless of factors such as lesion location or previous history of myocardial infarction [22]. Although previous IVUS-FFR studies demonstrated good diagnostic performance for detecting myocardial ischemia, their clinical use was limited due to the time-consuming nature of CFD calculations. Recently, a new method for fast computtaion of FFR from the fusion of IVUS and angiographic images was developed, allowing for accurate modeling of vessel bending geometry and inlet flow. Using this technique, AccuFFRivus, the real lumen of a 3D image can be obtained, and the true angle and direction of vessels presented by 2D angiography can be analyzed [7]. Previous studies demonstrated that AccuFFRivus had better diagnostic performance (93.75%) than DS% (65.62%) and MLA < 4 mm^2^ (53.12%). The diagnostic performance (accuracy 94.4%) and area under the curve (AUC) (0.960) were similar to those reported in previous studies. Therefore, coronary artery stenosis can be assessed using AccuFFRivus, which is a time-efficient and accurate method, and the visualized anatomic geometry of the coronary artery can be used for subsequent clinical planning and therapeutic regimens.

Although hemodynamic variables and anatomical geometry were not directly compared between CCTA and IVUS through this study, promising results were observed in the comparison of diagnostic performance between invasive FFR and IVUS-FFR or CT-FFR [23, 24]. However, these investigations did not compare the diagnostic efficacy and benefits of CT-FFR and IVUS-FFR. CT-FFR is used for quick capture of coronary anatomical and functional information non-invasively; however, it lacks the ability to identify lesion characteristics such as plaque load and high-risk plaque features [25]. IVUS-FFR, on the other hand, can assess the 3D morphology of coronary stenosis, providing lumen border and plaque features and accurately segmenting the lumen and exterior elastic membrane [7, 22]. Additionally, stent placement can be improved, and clinical outcomes may be enhanceed using IVUS-FFR. Thus, by combing the advantages of CT-FFR and plaque characteristics, IVUS-FFR allows for comprehensive assessment of coronary stenosis. AccuFFRct and AccuFFRivus were used in this study for hemodynamic assessment of target vessels without the need of pressure guidewire and hyperemic agents. The results demonstrated the high efficacy of both AccuFFRct and AccuFFRivus in diagnosing coronary artery stenosis and their potential as indexes to identify hemodynamic significance. Although both AccuFFRct and AccuFFRivus exhibited similar correlation and diagnostic performance with FFR, they serve different clinical roles. CT-FFR, known as the gatekeeper to the pathway of coronary angiography, was obtained from the outpatient setting and could reduce the number of unnecessary coronary angiography in patients without functionally significant CAD [26–29]. However, CT-FFR cannot assess complex lesion and plaque features [25, 28, 29]. AccuFFRivus had slightly better diagnostic performance and the ability to identify complex lesions and plaque characteristics, thereby guiding clinical diagnosis and revascularization procedures. Moreover, the AccuFFRivus only required 5 min per examination to calculate the virtual FFR. The physiological significance of coronary stenosis can be assessed immediately after IVUS image acquisition without the need for additional instrumentation. Therefore, IVUS-FFR enables easy and fast coronary anatomical and physiological evaluation of CAD in the cardiac catheterization laboratory. Additionally, stent malapposition and under-dilation can also be determined in patients with stents, which may potentially improve the accuracy of AccuFFRivus’ physiological assessment in the stented segments [7, 23]. Based on the advantages of IVUS-FFR, it is a “one-stop” assessment in the catheter room in the pre-percutaneous coronary intervention (PCI) and post-PCI evaluations, offering a novel technique to diagnose CAD based on intracoronary imaging.

However, our study had several limitations. First, it was a single-center and retrospective study with a small sample size. This might have introduced selection bias even though consecutive patients were included. The limited number of enrolled patients due to the low adoption rate of patients undergoing both IVUS and FFR in clinical practice also affected the statistical efficiency of the study. Secondly, AccuFFRct and AccuFFRivus were only assessed in the main coronary arteries, excluding side branches, which may have affected the diagnostic accuracy of AccuFFRct and AccuFFRivus and disregarded the impact of collateral stenosis on myocardial ischemia. Thirdly, AccuFFRct and AccuFFRivus computation requires automatic reconstruction of 3D anatomical models of coronary vessels, and further studies should be conducted to analyze the impact of anatomical features on diagnostic accuracy in target vascular lesions. Lastly, there are some practical limitations associated with the use of AccuFFRivus and AccuFFRct in the clinical setting. AccuFFRivus requires careful attention to the projection angle and location of the target lesions in coronary angiography, and AccuFFRct has a relatively complex reconstruction process, it is recommended that calculation be performed by well-trained staff to ensure accurate results.

## Conclusions

AccuFFRivus and AccuFFRct exhibit strong correlation and good agreement with invasive FFR, providing good diagnostic accuracy in detecting myocardial ischemia. IVUS-FFR has the potential to become a new clinically relevant tool for functional evaluation in diagnosing functional significance of CAD and may emerge as a mainstream technique for lesion-specific coronary assessment bedises CT-FFR.

## Methods

### Study population

A retrospective, single-center, observational study was conducted from January 2020 to July 2021, including consecutive patients with CAD who had the IVUS, FFR and 2D-QCA within six months of completing the CCTA were eligible for enrollment. The inclusion criteria were as follows: (1) age ≥ 18 years; (2) patients with suspected or known CAD; and (3) at least one lesion with 30–80% diameter stenosis (DS%) based on visual estimation. The exclusion criteria were as follows: (1) angiographic evidence of thrombi-containing lesions; (2) patients in which the IVUS catheter could not cross the lesion owing to tight stenosis or tortuosity; (3) severe valvular heart diseases; (4) left ventricle ejection fraction < 30%; (5) significant foreshortening or vessel overlapping; (6) previous coronary artery bypass grafting; (7) inadequate contrast flush; (8) deep catheter intubation into the lesion precluding complete visualization of stenosis; (9) severely calcified vessels; (10) balloon dilatation performed before IVUS; or (11) inconsistent image format.

The study was conducted in compliance with the Declaration of Helsinki, and was approved by the Ethics Review Committee of Zhejiang Hospital. Individual informed consent was waived due to the retrospective nature of the study.

### Image acquisition and data analysis

The present investigation was a retrospective, single-center, observational study performed at the Zhejiang Hospital. This study aimed to compare the diagnostic performance between AccuFFRivus and AccuFFRct in real-world clinical practice, using FFR as the reference standard. The same equipment, Siemens Force CT and Boston Scientific/SCIMED IVUS were used to perform imaging for all patients. The latest guidelines were used to conduct CCTA and IVUS procedures, respectively [7, 9, 10, 30, 31]. Two senior radiology physicians, blinded to the clinical data, independently analyzed all images to ensure unbiased analysis. In cases of disagreement, a third, more experienced radiologist (an associate or chief physician) reviewed the images for the final determination. The degree of stenosis was quantitatively assessed based on the criteria for segmented coronary vessel images. An obstructive coronary artery lesion was indicated when luminal diameter stenosis was greater than 50% [29, 32]. The baseline patient information, clinical data, and auxiliary examination results were collected and screened by researchers at the applicant’s hospital. To calculate AccuFFRct and AccuFFRivus, the CCTA and IVUS data were subsequently analyzed at the core laboratory of ArteryFlow Technology (Hangzhou, China). Then the group of Zhejiang Hospital analyzed the AccuFFRivus and AccuFFRct. To ensure accurate and high-quality data, both CTA and IVUS imaging were performed in strict adherence to standardized protocols with blinded and independent image analysis.

### ICA and measurement of physiological indices

The radiographic system Allura Xper FD20/10 (PHILIPS Medical Systems, the Netherlands) was used for the angiographic imaging at a rate of 15 frames/s. The contrast medium was injected at a stable rate of approximately 4 mL/s using a pump. The 2D-QCA was performed using the Angiogram QCA software (Allura Xper FD20/10; PHILIPS Medical Systems, The Netherlands). A coronary pressure wire (St. Jude Medical, St. Paul, Minnesota, USA) was used to calculate FFR with the pressure sensor positioned at 2–3 cm distal to the target lesion of the coronary artery. Before placement, the pressure wire was calibrated and equalized, and intravenous adenosine triphosphate concentration was 150–180 g/kg/min to induce maximum hyperemia of the coronary microvascular system. Simultaneously, the distal and proximal coronary artery pressures at the pressure sensor (Pd) and coronary ostium (Pa) were recorded. The pressure sensor was then pulled back to the proximal end to assess or correct pressure drift. The FFR was determined by dividing Pd by Pa. Further analysis was performed at the core laboratory using all ICA and FFR data. Thus, a standardized radiographic system, pressure wire, and software were used, and strict protocols were followed for data collection and analysis to ensure accuracy and reliability [33].

### AccuFFRct measurements

The CT-FFR results were calculated using the latest AccuFFRct analysis software (AccuFFRct, V 1.0, ArteryFlow Technology, Hangzhou, China) and analyzed in the AccuFFRct core laboratory.

The calculation process comprised the following four steps: (1) reconstruction and segmentation of a 3D model of the coronary artery and left ventricle using the CCTA image data. First, the fast marching algorithm and colliding fronts algorithm were applied to the aortic and coronary tree segmentation, and the level set method was used to identify the optimal vascular boundaries. Moreover, the Marching cubes method [34–37] was adopted to obtain the anatomical model of the coronary tree. Following this, the deep learning segmentation method based on an eight-layer residual U-Net was used to extract the left ventricular model and determine the myocardial volume [38–40]. (2) The coronary artery anatomical model was preprocessed, including hole inspection, smoothing, and boundary surface editing of the 3D model, and then transformed into a mesh model. A numerical CFD simulation was performed later to obtain the blood flow field. (3) The Navier–Stokes equations were solved using the finite volume method, and flow field information was calculated, including the pressure and velocity of each cell of the mesh model. (4) The AccuFFRct value was assessed as the ratio of the distal pressure at the FFR measuring point to the mean aortic pressure. Depending on the CT image quality, the AccuFFRct analysis required about 35 min per examination [12].

### AccuFFRivus measurements

The latest AccuFFRivus analysis software AccuFFRivus V1.0 (ArteryFlow Technology, Hangzhou, China), was used for analysis in the AccuFFRivus core laboratory. Firstly, ECG gating and geometric parameter calibration were performed using the radiographic angiography image processing [41–46], and an accurate vascular model was generated using two matched images obtained from two different projections at the end of the diastolic cardiac stage [47, 48]. This calibration method can eliminate geometric errors and achieve the optimal matching of two images using three pairs of physiological points. The vascular lumen boundary of the angiographic image was detected using Dijkstra minimum path algorithm [34, 35], and could be adjusted as required. The analysis was performed from the proximal segment points to the distal segment points, and finally, the whole vessel lumen point cloud with layered distribution was constructed. Based on the principle of minimum energy, the trajectory of the IVUS catheter in the vessel was calculated, and the position of the guide wire was extracted using the Dijkstra algorithm [49, 50].

The images at the end of the diastole were selected to process IVUS images, and the lumen of 2D IVUS images was automatically segmented using a U-Net-based algorithm [51, 52]. The IVUS and angiographic images were fused to conduct 3D coronary artery modeling. The blood flow velocity was determined from the geometric characteristics of the artery and the number of frames of blood flow from the proximal end to the distal end when the blood flow velocity was assumed to be directly proportional to the square of the coronary artery diameter. The reference vessel diameter was determined by the linear fitting of the initial reference vessel diameter slope. The pressure drop from the proximal to distal may be composed of friction-related viscous and expansion pressure drop related to the coefficient of energy loss and flow rate. The AccuFFRivus value is determined by dividing the mean distal coronary pressure by the mean proximal aortic pressure. Depending on IVUS image quality, the AccuFFRivus analysis required around 5 min per examination [7, 53, 54].

### Statistical analysis

Continuous and binary variables were presented as mean ± standard deviation (SD) and percentages, respectively. Pearson’s or Spearman’s correlation coefficients were used to quantify the correlations between AccuFFRivus, AccuFFRct, and invasive FFR. Bland–Altman plots were used to assess the agreements between AccuFFRivus, AccuFFRct, and invasive FFR, which displayed the differences between each pair of measurements versus their mean values with reference lines for the mean difference of all paired measurements. The agreement limits were defined as the mean ± 1.96 SD of the absolute difference. To predict functionally significant stenosis (defined as FFR ≤ 0.80), sensitivity, specificity, and the Youden index (defined as [sensitivity/100] + [specificity/100] − 1) were calculated for different cutoff values of AccuFFRivus and AccuFFRct. To assess the area under the curve (AUC) of AccuFFRivus and AccuFFRct, receiver operating characteristic (ROC) curve analysis was performed. All statistical analyses were performed using MedCalc (version 19.0, MedCalc Software, Ostend, Belgium), and *P* < 0.05 was defined as statistically significant.

## Data Availability

The data that support the findings of this study are available from the corresponding author upon reasonable request.
